# Measuring the impact of household energy consumption on respiratory diseases in India

**DOI:** 10.1186/s41256-019-0101-7

**Published:** 2019-04-18

**Authors:** Mohammad Ahmad Faizan, Ramna Thakur

**Affiliations:** School of Humanities and Social Sciences, Indian Institute of Technology Mandi, Kamand Campus, Himachal Pradesh 175075 India

**Keywords:** Solid fuels, Clean fuels, Cooking, Energy, Household, Respiratory diseases

## Abstract

**Background:**

Most households in developing countries like India are not able to afford to get the services of efficient energy for cooking and lighting. Therefore, they rely mostly on solid fuels (firewood, dung cakes, crop residue, coal/coke/lignite). Such fuels cause respiratory diseases like tuberculosis, asthma respiratory cancer. Hence, this study aims to estimate the association between different types of energy used and the prevalence of respiratory diseases in India where more than 50% of the population relies on solid fuels for cooking.

**Methods:**

The study is based on 117,752 respondents who were diagnosed with various chronic diseases such as diabetes, chronic heart diseases, leprosy, chronic renal diseases, tuberculosis, asthma etc. from District Level Household Survey (DLHS-4) which was conducted in 2012–13. Individuals who were diagnosed with a chronic illness after a proper medical examination have been considered as a dependent variable. Exposure to the type of cooking fuel is the main exposure variable, which recognises the dependence on energy. Logistic regression has been utilized to understand the association between the use of solid fuels for cooking and the prevalence of respiratory diseases.

**Results:**

The dependence on solid fuels is very high in rural areas (72.22%) as compared to urban areas (21.43%). Among different castes, the reliance on solid fuels for cooking is highest among Scheduled Castes (61.79%) and Scheduled Tribes (70.46%). Individuals living in households where crop residue and coal/lignite is used for cooking suffer from asthma/chronic respiratory failure in the higher proportion as compared to others. Results further revealed that the use of solid fuels for cooking has a strong association with respiratory diseases. Individuals living in households where solid fuels like firewood [OR: 1.27 (0.001); C.I.: 1.19–1.35], crop residue [OR: 1.33 (0.001); C.I.:1.19–1.48], and coal [OR: 1.60 (0.001); C.I.:1.32–1.93] are used as primary fuel for cooking are 17 to 60% more likely to suffer from respiratory diseases.

**Conclusion:**

Use of solid fuels is associated with respiratory diseases like asthma, tuberculosis and cancer of the respiratory system. Assuming these associations are causal, therefore, about 17 to 60% of the respiratory diseases in India could be prevented by providing access to clean cooking fuel to the individuals.

## Background

Household energy consumption as a concept focuses mainly on the energy used for cooking, washing clothes, lighting houses, heating and cooling, running appliances, etc. [1] According to World Health Organization (WHO), three billion people (more than 40% of global population) are dependent on solid fuels like firewood, dung cakes, coke, coal, and agricultural residues around the globe. This is imposing severe challenges especially to low and middle-income countries [2] where many households do not have the means to satisfy their basic energy needs [3]. Access to clean energy for household consumption is an important aspect of national and global progress. People who lack access to clean energy are deprived of an opportunity to lead a healthy life [4]. It is linked with food, clean water, education, health, and hygiene, which are important indicators of the development [5, 6].

The dependence on solid fuels results in various negative externalities which are sometimes life-threatening [7]. In developing countries, most of the households use traditional stoves for their cooking and heating needs [8]. These stoves emit smoke which causes acute respiratory illness and even cancer, where mostly women and children are at the receiving end [9]. According to WHO (2016), more than 4 billion deaths occurred due to the household air pollution around the world which were mostly in the low and middle-income countries. In 2016, 56.9 million deaths were recorded, including 6 million deaths due to chronic pulmonary obstructive disease (COPD) and acute respiratory infection (ARI) which is among the top ten causes of deaths around the world. More than 9 million deaths were due to ischemic heart diseases (IHDs), and 1.3 million deaths were due to Tuberculosis (TB) [10]. Various studies carried out in African countries found that indoor air pollution and acute respiratory infections have a strong relation and acute respiratory infection is said to be the principal cause of students being absent in the schools [11].

In India, more than 75% rural and about 25% urban population use solid fuels as their primary source of energy for cooking. In rural areas, the dependence on firewood and chips is staggering 67%, and nearly 10% rely on dung cakes as their primary source of cooking. According to GoI [12] report on medical certification of causes of deaths, 9% of medically reported deaths are due to diseases of respiratory systems in the country. Among the conditions of the respiratory system, pneumonia and asthma cause 21.1% and 8.8% of deaths respectively.

Energy security and climate change have been given extensive coverage in the literature, but attention towards household energy consumption and its impact on health is very minimal. In 2015, along with the adoption of the 2030 agenda for sustainable development, 17 sustainable development goals were also adopted. One of these goals is to ensure “*good health and well-being”,* and another is *“access to affordable, reliable, sustainable and modern energy for all.”* Understanding the linkages between good health and well being with sustainable energy, offers insights into how energy consumption contributes towards diseases of the respiratory system [13–20].

In India, many studies have looked into the association between various respiratory diseases and the use of solid fuels. Most of these studies have looked at a specific respiratory disease like TB [21–27], ARI [28–31], COPD [32–34]. Most of these studies are based on a specific region with a limited size of sample apart from a study conducted by V. Mishra and associates [22]. The conclusions drawn in these studies with relatively small samples are limited by high levels of heterogeneity.

By reflecting on the above issues, we report findings on solid fuel use for cooking and its association with respiratory diseases (asthma, TB and respiratory cancer as reported in the survey) among 1.6 million individuals from 21 states in India from DLHS-IV survey. A better understanding of the use of a type of fuel for cooking and the incidence of respiratory diseases will help to analyse the association between these two at the national level.

## Methods

District Level Household Survey (DLHS-4) which was conducted in 2012–13 has been utilized in this study. The survey has covered 21 States and Union Territories of the country to collect firsthand information. The multi-stage stratified sampling design was adopted which covered 378,487 households consisting of 1,687,736 individuals. Only those respondents who were diagnosed with various chronic diseases like diabetes, chronic heart disease, chronic liver disease, anaemia etc. are included in the analysis. 144,880 individuals had responded that they had symptoms about the illness which persisted for more than one month. Among these individuals who seek medical care, 118,618 were diagnosed with any chronic diseases. Individuals were asked about the type of fuels used for cooking in their Houses and individuals which had responded with “other”, and “no cooking” are excluded from the analysis. Also, those individuals who don’t have any cooking arrangements in their houses are excluded.

After excluding the missing values and dropped observations, a total of 117,752 respondents were included for final analysis. Table 1 provides the basic demographic profile of the sample population. A total of 378,487 households were surveyed in this survey which includes 1,687,736 individuals. Among the total population, Hindus consist of more than 67% followed by Christians and Muslims. Details of the sampling design, survey tools, and data collection methods are provided in the survey report [35]. The survey collected the information using four questionnaires; facility questionnaire, household questionnaire, village questionnaire, and women questionnaire.

**Table 1:** Demographic characteristics of the sample population

Variables		Total
Sample Households		378,487
Sample Individuals		1,687,736
Religion	Hindu	1,156,695
	Muslim	155,343
	Christian	192,777
	Sikh	108,918
	Others	72,011
Age	0–5	149,199
	6–14	270,794
	15–59	1,079,947
	60+	181,123
Caste	General	359,707
	Scheduled Caste	377,942
	Scheduled Tribe	305,484
	Other Backward Castes	556,358
Sex	Male	848,433
	Female	837,930
	Others	242
Household Structure	*Pucca*	766,153
	Semi*-pucca*	602,398
	*Kaccha*	308,504
	Others	8241
Standard of Living	Low	592,509
	Medium	575,026
	High	496,606
Locality	Rural	1,004,130
	Urban	660,011

*Note:* *The totals of all different variables in this table are not matching due to the missing values in each category*

### Health outcome and exposure variable

**Fig. 1:**
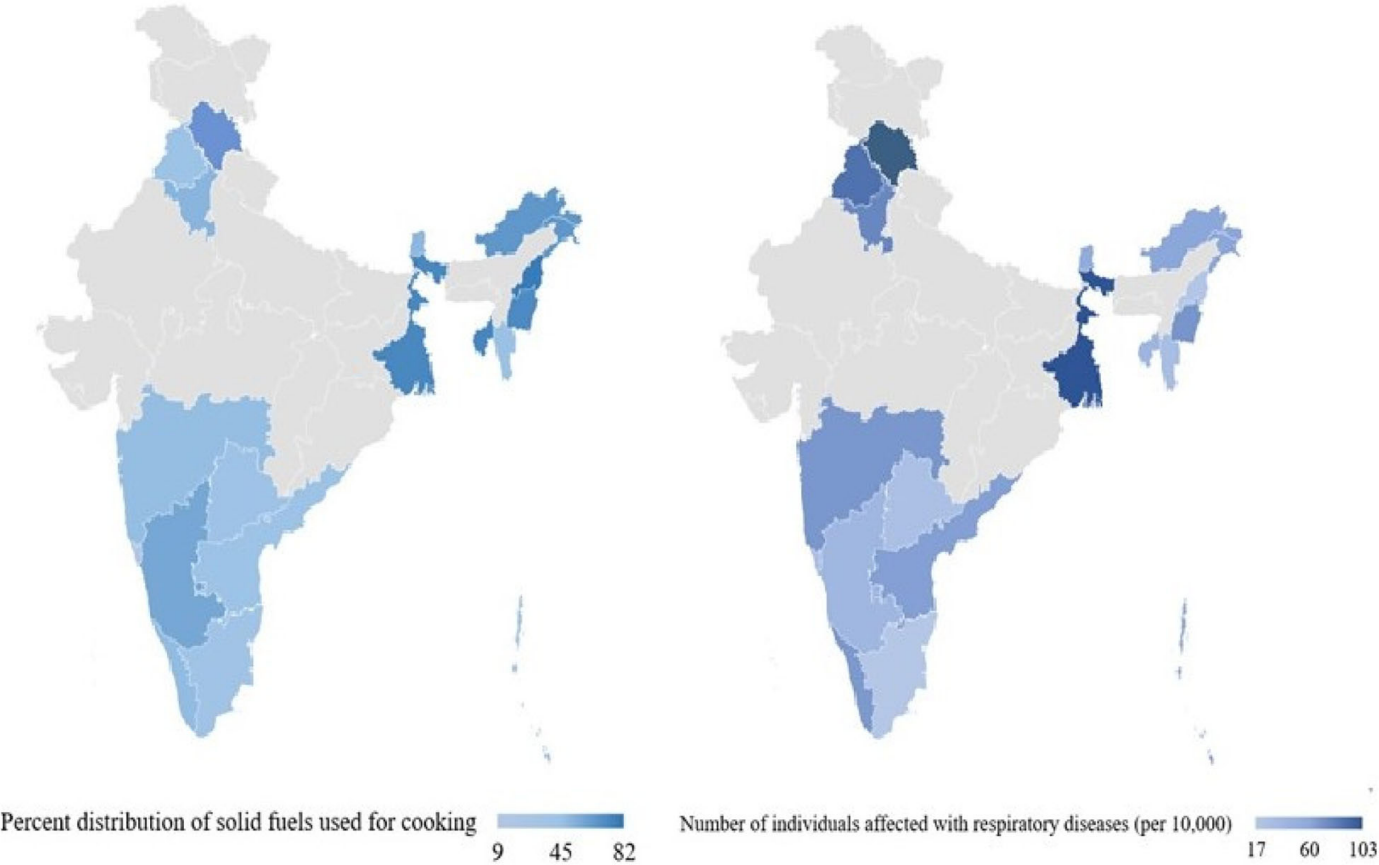
Percent distribution of solid fuel users (left) and percent distribution of individuals suffering with respiratory diseases (right) in the study areas

**Fig. 2:**
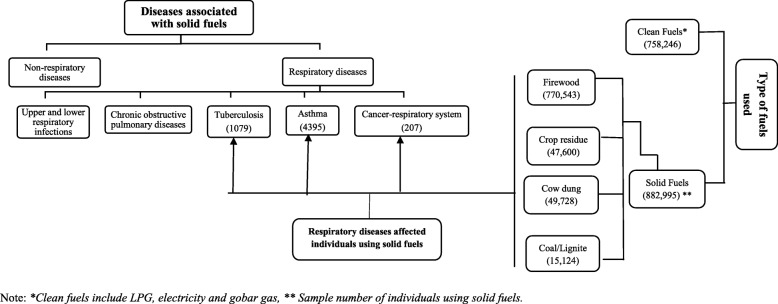
Diagrammatic representation of the association of respiratory diseases and solid fuels used for cooking in the study areas

Diseases which affect the airways and other structures of the lung are known as respiratory diseases.^1^ Chronic obstructive pulmonary disease (COPD), asthma, occupational lung diseases and pulmonary hypertension are few examples of the respiratory diseases. This study includes asthma, tuberculosis and cancer of the respiratory system, as in DLHS-4 data is available on these three diseases only in respiratory disease category. Respondents were asked questions regarding the chronic illness during the last one year and whether they sought any medical care. If respondents sought medical care, then the question was asked ‘what was diagnosed by the doctors’? Only those individuals who were diagnosed with any chronic diseases are considered as dependent variables in this study. The outcome variable is a dichotomous; 0 for diseases other than respiratory diseases and 1 for respiratory diseases. Exposure to the type of cooking fuel has been recognized as the dependence on either clean or solid fuels. The cooking fuel was coded as firewood, crop residue, cow dung, coal/lignite, liquefied petroleum gas (LPG), electricity and kerosene Figs. 1 and 2.

### Covariates

Various socio-economic variables have been considered as covariates, as the association between energy use and chronic respiratory diseases can be confounded. Place of residence, the standard of living, religious groups, caste, type of locality, the household structure, cooking arrangements in-house, and source of lighting. Standard of living index as a proxy for socio-economic status was calculated based on household amenities, such as the source of drinking water, type of household, lighting source, toilet facility, and ownership of goods. The details of the scoring and classification into low, medium and high categories are given in DLHS-2, followed in DLHS-4 as it is [35] and have been included in the analysis.

### Analysis

With the help of Stata 13 Adjusted logistic regression has been used to explain the relationship between respiratory diseases and energy consumption. A meaningful interpretation of the results is made through odds ratio. The odds ratio is obtained by taking antilog of different slope coefficients.

## Results

Table 2 describes the basic socio-economic characteristics of the individuals by the source of energy used for cooking. Among the high standard of living, 84% use clean fuels like LPG and electricity as a primary source of energy for cooking. While 90% of the individuals in the low standard of living households use solid fuels as a primary source of energy for cooking. Among different religious groups, 52% Hindus and an equal percentage of Muslims, 64 and 54% of Christians and Sikhs respectively use solid fuels as a primary source of energy for cooking. Overall, the reliance on solid fuels for cooking is more than 50% among different religious groups. Individuals belonging to socially marginalized households like scheduled castes (SCs) and scheduled tribes (STs) use 64 and 71% solid fuels for cooking respectively. There is a huge gap between urban and rural households using solid fuels. Nearly three-fourth of the population in rural households use solid fuels, and one-fourth of the total population in urban households use solid fuels as a primary source of energy for cooking (see Table 2). Table 3 shows the number of individuals affected by respiratory diseases per hundred thousand by the type of fuels used for cooking. 875 and 780 per hundred thousand individuals suffer from asthma or chronic respiratory failure who belong to those households where crop residue and coal/lignite is used as the primary fuel for cooking. On the other hand, 650 individuals per hundred thousand suffer from asthma or chronic respiratory which use cow dung. Similarly, 193 and 174 individuals per hundred thousand suffer from TB using cow dung cakes and crop residues as the primary fuel for cooking. 42 and 39 individuals per hundred thousand suffer from cancer of respiratory system who use kerosene and crop residues as primary fuel for cooking. Table 3 clearly shows that crop residues, cow dung and coal & lignite are the fuels which significantly affect the health of the individuals.

**Table 2:** Percent distribution of the source of energy for cooking among different socioeconomic groups

	Sources of energy for cooking							
Variable	LPG/Electricity	Firewood	Crop residue	Cow dung	Coal/Lignite	Kerosene	No Cooking	Others
Standard of Living^a^								
High	83.93	11.28	1.69	2.59	0.19	0.24	0.04	0.04
Middle	54.87	36.52	2.74	3.10	0.79	1.74	0.17	0.07
Low	10.19	79.44	4.21	2.74	1.21	1.68	0.35	0.17
Religious groups								
Hindus	47.97	43.97	3.17	2.49	0.78	1.31	0.22	0.10
Muslims	48.29	38.78	5.57	2.75	1.73	2.34	0.23	0.32
Christians	35.76	62.30	0.27	0.14	0.42	1.01	0.08	0.02
Sikhs	46.27	33.53	4.83	13.85	0.54	0.65	0.28	0.04
Others^b^	36.36	61.41	0.49	0.29	0.40	0.92	0.10	0.03
Caste								
Gen	59.22	30.64	3.72	3.72	1.04	1.23	0.23	0.20
SC	36.35	50.86	4.65	5.24	0.93	1.62	0.24	0.11
ST	28.57	67.96	1.14	0.85	0.47	0.87	0.09	0.04
OBC	52.96	40.82	2.30	1.83	0.52	1.28	0.23	0.06
Locality								
Urban	76.09	18.69	0.86	0.78	1.03	2.21	0.27	0.06
Rural	26.89	63.00	4.37	4.11	0.62	0.72	0.16	0.12

^a^*Standard of Living (SLI) was calculated as per the method provided in the DLHS-II. There were 24 household amenities included in the construction of SLI. Few of the amenities, for example, were ownership of refrigerator, car, fan, telephone, washing machine, TV, tractor, and water pump,* etc.

^b^ *Others includes Jains, Buddhists, Jewish, Zorastrians, Atheists, etc*

**Table 3:** Individuals affected by the respiratory diseases among different groups based on the type of fuel used for cooking (Per hundred thousand)

Type of fuel used for cooking	Asthma/Chronic respiratory failure	Tuberculosis	Cancer-respiratory system
LPG/Electricity	392	65	22
Firewood	456	112	23
Crop Residue	875	174	15
Cow Dung Cake	650	193	39
Coal/Lignite	780	116	28
Kerosene	489	108	42

*Note: Figures are based on authors’ calculation from DLHS-IV*

In the above tables, it is evident that the reliance on solid fuels for cooking is more than 50%. Individuals belonging to socially and economically marginalized households are more dependent on the use of solid fuels for coking (see Table 2). Similarly, it is apparent that the individuals belonging to those households where solid fuels are used as a primary source for cooking suffer more from various respiratory diseases. To find out the association between the respiratory diseases and solid fuel use, we have used logistic regression. The results of the logistic regression showing the association between the type of fuel used for cooking and respiratory diseases after controlling other factors are given in Table 4. Individuals who are suffering from the chronic respiratory diseases like asthma, TB, and respiratory cancer were coded in a binary outcome, where “0” means diseases other than respiratory disease and ‘1’ represents the individuals suffering from respiratory disease. Type of cooking fuel use, the standard of living index, religious groups, caste, locality, household structure, arrangement for cooking and source of lighting have been included in the logistic model. Type of fuel used for cooking with firewood [Odds Ratio (OR) 1.26; CI 1.19–1.35], crop residue (OR 1.33; CI 1.19–1.48), cow dung (OR 1.17; CI 1.04–1.3), coal (OR 1.60, CI 1.32–193) and kerosene (OR 1.40; CI 1.14–1.71) have a significant association with respiratory diseases. Furthermore, kerosene used as a source of lighting has a considerable association with respiratory diseases (OR 1.18; CI 1.05–1.33) in India.

**Table 4:** Adjusted logistic regression of the socio-demographic factors and respiratory diseases

Chronic Respiratory Disease	Adjusted logistic regression		
	Odds Ratio	*P* Value	Confidence Interval at 95%
Cooking Fuel used			
LPG/Electricity/Biogas	1.00		
Firewood	1.27	0.001	1.19–1.35
Crop Residue	1.33	0.001	1.19–1.48
Cow Dung Cake	1.17	0.007	1.04–1.31
Coal^**^	1.60	0.001	1.32–1.93
Kerosene	1.40	0.001	1.14–1.71
Standard of Living Index			
High	1.00		
Middle	1.24	0.001	1.16–1.32
Low	1.46	0.001	1.34–1.58
Religion			
Hindu	1.00		
Muslim	1.13	0.003	1.04–1.22
Christian	0.97	0.568	0.89–1.07
Sikh	1.28	0.001	1.18–1.38
Others	1.09	0.109	0.98–1.21
Caste			
Others	1.00		
SCs	1.07	0.032	1.00–1.14
STs	1.24	0.001	1.14–1.35
OBCs	0.96	0.144	0.90–1.01
Locality			
Urban	1.00		
Rural	1.08	0.003	1.03–1.14
Household Structure			
*Pucca*	1.00		
Semi*-pucca*	1.09	0.002	1.03–1.16
*Kachha*	1.18	0.001	1.09–1.28
Other	0.95	0.763	0.66–1.35
Cooking Arrangement			
Kitchen Inside-Cooking	1.00		
No-Kitchen Inside-cooking	1.09	0.016	1.02–1.18
Kitchen Outside-cooking	1.07	0.056^*^	1.00–1.14
No Kitchen Outside Cooking	1.05	0.381	0.94–1.16
Source of Lighting			
Electricity/Solar	1.00		
Kerosene	1.18	0.005	1.05–1.33
Other-oils	0.84	0.261	0.62–1.13

*Note: Figures are based on authors’ calculation from DLHS-IV* ^*^ *Significant at 10%* ^**^ *includes coke and lignite*

It is also evident from the results that individuals with a low standard of living are more likely to suffer from respiratory (OR 1.46; C. I 1.34–1.58) as the majority of the individuals belonging to the low standard of living households are using solid fuels. Results show that as the standard of living increases, the chances of having respiratory diseases decline. Results of different religious groups show that Muslims (OR 1.13; C. I 1.04–1.22) and Sikhs (OR 1.28; C. I 1.18–1.38) use more of solid fuels and are having significantly higher chances of respiratory diseases. Results also show that individuals who reside in rural areas (OR 1.08; CI 1.03–1.14) use more of solid fuels and are having significantly higher chances of respiratory diseases as compare to urban areas. Also, those individuals who reside in *Kaccha* house (OR 1.18; C.I. 1.09–1.28) and Semi-*pucca* houses (OR 1.09; C.I. 1.03–1.6) have significant chances of higher respiratory diseases as compared to the individuals who reside in *pucca* houses. Individuals who reside in the households where food is cooked inside the house without having kitchen also have significantly higher chances of respiratory diseases (OR 1.09; CI 1.02–1.18).

## Discussion

Solid fuel as a source of cooking is primarily used in low and middle-income countries [36] where availability and affordability of the clean energy is still confined to the richer sections of the society. Solid fuels release a lot of pollutants like carbon monoxide (CO), carbon dioxide (CO_2_), sulfur dioxide (SO_2_), nitrogen dioxide (NO_2_), volatile organic compounds (VOCs) or hydrocarbons (HC). Particulate matter of PM_10_ and PM_2.5_ are also released which are the primary causes of respiratory diseases. Emissions from the solid fuel combustion produces indoor air pollution which cause nearly 4 million premature deaths mainly in low and middle-income countries [37].

Economically and socially vulnerable sections of the society like poor, SCs and STs in India are more likely to suffer from the respiratory diseases as the majority of them rely on the solid fuels for cooking. In spite of substantial progress since independence, these sections are still deprived of having access to clean fuels. They rely mostly on solid fuels for cooking and live in the areas which are isolated, remote and ghettoized [38].

The wider gap in the use of clean fuels among rural and urban areas [17] has a significant effect on the outcome of respiratory diseases. The dependence on solid fuels in rural and urban areas is more than 75% and 20% respectively which results in the higher prevalence of chronic respiratory diseases in rural areas as compared to urban areas [39].

Majority of the toxins which are released using solid fuels are more harmful in the poorly ventilated houses. Semi *pucca* and *kaccha* houses mostly have a common kitchen which is either attached to the living room, or they have a single room which is used as a living room as well as kitchen. Individuals residing in these households having a kitchen inside have a significantly higher prevalence of respiratory diseases. Most of the young and older people occupy these areas and get exposed to higher levels of smoke [40]. The average family size in India is five members [41], and as per a report by the Ministry of Home Affairs, GoI [42] more than 75% of the households are having two rooms or less. In this situation, one can imagine, where they cook and where they sleep.

According to World Bank data, one in five Indians is poor and mostly relies on the solid fuels which make them more susceptible to health problems. They cannot afford the necessities which are required to live a healthy life. Poor people have to spend a bigger share of their total consumption expenditure on food, fuel, and light [43]. With an increase in income, it is expected that the households switch over from solid fuels to clean fuels like LPG and electricity [44].

There is a significant concern regarding the accessibility and the affordability of energy for cooking especially in the rural areas in the developing countries like India [45]. To address the huge gap between rural and urban households consumption of clean fuels, the government of India introduced Rajiv Gandhi Gramin LPG Vitaran Yojana, (RGGLV) in 2009. This scheme was launched to increase the penetration of LPG to cover the low potential rural areas. Furthermore, in 2016, the government of India launched another scheme called Pradhan Mantri Ujjwala Yojana (PMUY) to distribute five crore LPG connections to poor women free of cost by March 2019. This scheme aimed mainly to safeguard the health of women and children in the country.

Also, the government of India provides subsidy on Liquefied Petroleum Gas (LPG) to all households to decrease the household air pollution and curb the adverse health impacts. Though, LPG is a subsidized fuel in India, but is used largely by the advantaged groups [46]. Further, to deal with this issue and to support low-income households to transition to clean fuels, the government of India constituted a committee in 2010 [47]. As per the recommendations of the Committee, the subsidy to LPG consumers is transferred directly into the bank account of the consumers. In spite of the above mentioned efforts by the government of India, the use of solid fuels especially in rural areas is still a matter of concern. A sharp rise in the price of LPG in the past few years could be one of the factors of hindering people from its use. Another barrier to LPG penetration could be the high initial cost of the connection which includes the cost of the LPG cylinder and cook stoves [48–51]. While under PMUY scheme, the government provides the initial cost of the LPG cylinder, but the high cost of accessories and recurring cost of cylinder refill discourage poor consumer [48].

Access to efficient and safe energy is crucial for human development as well as for the overall development of the nation. There is a need to improve health education and bring cultural changes and switch over to clean or efficiency fuels for cooking. Over the years the Government of India has intervened to promote the use of clean fuel for cooking among households, but there is still a long way to go to increase the level of penetration. The rising prices of the LPG need to keep in check, and the government must ensure that the subsidized LPG reaches to the potential beneficiaries. To fulfil the three objectives of UN’s “Sustainable Energy for All” which are electricity, clean cooking and heating systems, the government must ensure the overcome of the barriers which are creating obstacles at the implementation level.

### Limitations of the study

To gather information regarding health is important in any health system but information related to health is not ample in low- and middle-income countries like India. The District Level Household Survey (DLHS) was launched in 1996–97 by the Government of India in response to the need for district-level data on Reproductive and Child Health Programme. This data is looking into the child and maternal health primarily, but some information related to major diseases have also been collected in the survey. The data lacks information on, inappropriate development of lungs along with physical activity, passive smoking, time spent in kitchen etc. which could have made the study more explanatory. There is a need of the holistic data sources which can help researchers to explore the association between the types of fuels used for cooking and respiratory diseases in India.

## Conclusion

Use of solid fuels is associated with respiratory diseases like asthma, tuberculosis and cancer of the respiratory system. Results of the study are consistent with the existing literature on other developing countries. High proportion of the individuals exposed to solid fuels account for high likeability of having respiratory diseases in the case of rural areas and the individual belonging to socially and economically marginalized groups. Assuming these associations are causal, hence, about 17 to 60% of the respiratory diseases in India could be prevented by providing access to clean cooking fuel to the individuals.

## Abbreviations

ARI: Acute Respiratory Infection
CO: Carbon Monoxide
CO_2_: Carbon Dioxide
COPD: Chronic Pulmonary Obstructive Disease
DLHS: District Level Health Survey
GoI: Government of India
HC: Hydrocarbons
IHD: Ischemic Heart Diseases (IHD)
IIPS: International Institute for Population Sciences
LPG: Liquefied Petroleum Gas
NO_2_: Nitrogen Dioxide
NSSO: National Sample Survey Organization
OR: Odds Ratio
PM_10_ & PM_2.5_: Particulate Matter 10 & Particulate Matter 2.5
PMUY: Pradhan Mantri Ujjwala Yojana
RGGLV: Rajiv Gandhi Gramin LPG Vitaran Yojana
SC: Scheduled Caste
SO_2_: Sulfur Dioxide
ST: Scheduled Tribe
TB: Tuberculosis
UN: United Nations
VOCs: Volatile Organic Compounds
WHO: World Health Organization

## Footnotes

1
https://www.who.int/respiratory/en/
